# Editorial: Women in drugs outcomes research and policies: 2021

**DOI:** 10.3389/fphar.2022.1059422

**Published:** 2022-11-14

**Authors:** Filipa Alves da Costa, Johanna Catharina Meyer, Dalia Dawoud

**Affiliations:** ^1^ Faculty of Pharmacy, University of Lisbon, Lisbon, Portugal; ^2^ Department of Public Health Pharmacy and Management, School of Pharmacy, Sefako Makgatho Health Sciences University, Pretoria, South Africa; ^3^ National Institute for Health and Care Excellence, London, United Kingdom; ^4^ Faculty of Pharmacy, Cairo University, Giza, Egypt

**Keywords:** gender equality, medicines access, cost-effectiveness, health technology assessement (HTA), medicines use, clinical trials

Science and gender equality are essential to ensure sustainable development. However, according to UNESCO, less than 30% of researchers worldwide are women. This Research Topic from Frontiers in Pharmacology aims to highlight research developed by female scientists whose line of work focuses on research, practice, and policy regarding access to cost-effective medicines and healthcare services. We have selected five articles to include in the Research Topic namely one opinion piece, one review article and three original research articles.

To a certain extent the collection aims to mimic the drug cycle, from the development and clinical trial stage to market entry until drug use, covering prescribing, dispensing and drug utilization by patients.

The opinion piece by Sarri focuses on the value of real-world evidence to inform health-care decisions. It provides a good overview of how data that are generated in a real-world environment can offer complementary evidence to randomized clinical trials, particularly by offering additional information such as the long-term effects of technologies, but also by leveraging clinicians’ expectations concerning the real risk-benefit ratio of these technologies when used in non-targeted populations. The author also challenges us to reflect on the role of real-world designs used to collate evidence about the experiences faced by disadvantaged populations and the extent to which barriers in access to healthcare may impact on the true value of technologies. The expansion of traditional cost-effective models is proposed to quantify health equality impacts.

Zhao et al. analyzed 72 registered clinical drug trials on pregnant women in China, with a focus on three factors namely osteoporosis, cancer, and assisted reproduction. The study highlights the significant shortage of safety trials for pregnancy-related drugs. The Research Topic aims to mimic the drug cycle; from the development of clinical trials to market entry as well as drug use, covering prescription, dispensing, and patient utilization. The findings in this original research reinforce the points presented by Sarri regarding the need for real-world evidence to overcome information gaps related to medicines at the market approval stage.

Daly et al. reviewed the economic models in NICE’s Technology Appraisals and used therapies for diabetes as an example. Through the analysis of ten Technology Appraisals pertaining to the treatment of diabetes, the authors concluded that there is a scarcity of robust data informing risk equations to enable derivation of transition probabilities. Although economic models are essential to support market entry decisions, key structural and methodological issues often impede or limit their value. HTA, a component of re-appraisal, seems therefore essential to base reimbursement decisions on, making use of real-world evidence that may be generated after market entry.

Moreira et al. explored the evolution of chemotherapy over 13 years, reporting a 50% increased use; the highest contribution was from the use of oral formulations and by the approval of 40 new anti-cancer medications. Using an alternative metric to drug consumption, the authors explored the various impacts on specific cancer sites, highlighting non-small-cell lung cancer as an important driver of costs, along with innovative therapies for advanced breast cancer. The authors conclude that the growing use of oral chemotherapy calls for investment to support patients in managing both medication adherence and adverse events, which require changes in healthcare system organization.

Aspects regarding health care functioning, the importance of interprofessional collaboration, and health information sharing platforms are introduced by Costa et al.. The authors report on the cost-effectiveness and cost-utility of a collaborative (involving pharmacies and primary care facilities) care model to manage hypertension and hyperlipidemia. Although the probability of the intervention to be cost-effective is below 30%, the innovative nature of the model established calls for additional research to create more sustainable solutions for primary health care. A person-centered approach prioritizing investment in primary care aligns with the conclusions drawn by Moreira et al.. The Astana declaration, signed in 2018 following the Global Conference on Primary Health Care, cites action to strengthen primary health care as the most inclusive and efficient approach to peoples’ health and wellbeing (World Health Organization, 2018). This will be essential for universal health coverage and reaching the Sustainable Development Goals.

The high quality of the published papers in this Research Topic demonstrates the capabilities of female researchers in this field, indicating that more support should be available to them to continue their valuable contributions.
